# Out of the dark: transitional subsurface-to-surface microbial diversity in a terrestrial serpentinizing seep (Manleluag, Pangasinan, the Philippines)

**DOI:** 10.3389/fmicb.2015.00044

**Published:** 2015-02-19

**Authors:** Kristin M. Woycheese, D'Arcy R. Meyer-Dombard, Dawn Cardace, Anacleto M. Argayosa, Carlo A. Arcilla

**Affiliations:** ^1^Department of Earth and Environmental Sciences, University of Illinois at ChicagoChicago, IL, USA; ^2^Department of Geosciences, University of Rhode IslandKingston, RI, USA; ^3^Institute of Biology, University of the Philippines DilimanQuezon City, Philippines; ^4^National Institute of Geological Sciences, University of the Philippines DilimanQuezon City, Philippines

**Keywords:** serpentinization, Zambales, deep subsurface biosphere, Illumina 16S rRNA, carbonate precipitation, hydrogen oxidation

## Abstract

In the Zambales ophiolite range, terrestrial serpentinizing fluid seeps host diverse microbial assemblages. The fluids fall within the profile of Ca^2+^-OH^−^-type waters, indicative of active serpentinization, and are low in dissolved inorganic carbon (DIC) (<0.5 ppm). Influx of atmospheric carbon dioxide (CO_2_) affects the solubility of calcium carbonate as distance from the source increases, triggering the formation of meter-scale travertine terraces. Samples were collected at the source and along the outflow channel to determine subsurface microbial community response to surface exposure. DNA was extracted and submitted for high-throughput 16S rRNA gene sequencing on the Illumina MiSeq platform. Taxonomic assignment of the sequence data indicates that 8.1% of the total sequence reads at the source of the seep affiliate with the genus *Methanobacterium*. Other major classes detected at the source include anaerobic taxa such as Bacteroidetes (40.7% of total sequence reads) and Firmicutes (19.1% of total reads). *Hydrogenophaga* spp. increase in relative abundance as redox potential increases. At the carbonate terrace, 45% of sequence reads affiliate with *Meiothermus* spp. Taxonomic observations and geochemical data suggest that several putative metabolisms may be favorable, including hydrogen oxidation, H_2_-associated sulfur cycling, methanogenesis, methanotrophy, nitrogen fixation, ammonia oxidation, denitrification, nitrate respiration, methylotrophy, carbon monoxide respiration, and ferrous iron oxidation, based on capabilities of nearest known neighbors. Scanning electron microscopy and energy dispersive X-ray spectroscopy suggest that microbial activity produces chemical and physical traces in the precipitated carbonates forming downstream of the seep's source. These data provide context for future serpentinizing seep ecosystem studies, particularly with regards to tropical biomes.

## Introduction

Deep subsurface habitats are critical to the evolution and sustainability of life on Earth. These habitats have potentially served as refugia from mass extinction events numerous times throughout Earth's history. Multiple examples of hydrothermal systems supporting the chemolithotrophic growth of thermophilic and hyperthermophilic archaea have been documented from the subseafloor (Amend and Teske, 2005). Studies of microbial community dynamics in subseafloor sediments suggest that symbiotic relationships occur between sulfate reducing bacteria and ammonia-oxidizing archaea in the Miscellaneous Crenarchaeotal Group (MCG) (Amend and Teske, 2005). The oligotrophic nature of these environments likely fuels such symbioses out of necessity.

The deep terrestrial subsurface is also proposed to support a vast biosphere, where total cell counts range from 3.5 × 10^30^ to 2.9 × 10^20^ (Whitman et al., 1998; Kallmeyer et al., 2012). The release of hydrogen gas (H_2_) from diverse geologic processes may support a vast H_2_-based deep subsurface biosphere (Nealson et al., 2005). Serpentinization (i.e., the low-temperature hydrous alteration of olivine and pyroxene) is known to produce volumes of H_2(g)_ and CH_4(g)_ (Cardace and Hoehler, 2009; Marlow et al., 2014) via the following reactions:

(1) $$\begin{array}{l} {\left(\text{Fe},\text{Mg}\right)}_{2}{\text{SiO}}_{4}+{\text{H}}_{2}{\text{O}}_{\left(\text{l}\right)}\to {\left(\text{Fe},\text{Mg}\right)}_{3}{\text{Si}}_{2}{\text{O}}_{5}{\left(\text{OH}\right)}_{4}\\ \;+\;\left(\text{Mg},\text{Fe}\right){(\text{OH})}_{2}+{\text{Fe}}_{3}{\text{O}}_{4}+{\text{H}}_{2\left(\text{g}\right)} \end{array}$$

(2) $${\text{4H}}_{2(\text{g})}+{\text{CO}}_{2(\text{g})}\to {\text{CH}}_{4}(\text{g})+2{\text{H}}_{2}{\text{O}}_{\left(\text{l}\right)}$$

The highly exothermic serpentinization reaction (Equation 1) proceeds as olivine becomes hydrated by meteoric water to produce serpentine, brucite, magnetite, and hydrogen gas. Methane production becomes thermodynamically favorable under these circumstances (Equation 2), and may be catalyzed abiotically (i.e., Fischer-Tropsch Type, or FTT, synthesis) or via biological methanogenesis (Schrenk et al., 2013). Isolated from the atmospheric reservoir, carbon dioxide (CO_2_) becomes limited in the deep subsurface system. Exposure to atmospheric CO_2_ at the terrestrial surface results in precipitation of calcium carbonate (CaCO_3_) from the alkaline, calcium-rich fluid:

(3) $${\text{Ca}}^{+2}+{\text{CO}}_{3}^{-2}\to {\text{CaCO}}_{3}$$

As precipitation occurs along the outflow channel, CaCO_3_ crystals may entomb biological material and geochemically preserve microbial activity. Analysis of carbonates associated with serpentinizing seeps may therefore provide insight for the interpretation of analogous fossil systems on Earth (Blank et al., 2009).

The purpose of this study is to characterize the microbial community along the outflow channel of a terrestrial serpentinizing seep via high-throughput 16S rRNA gene amplicon sequencing analysis, and determine the effects of surface exposure on microbial diversity. Manleluag Spring National Park is located in the Pangasinan province, the Philippines. The park hosts several known serpentinizing seeps originating in a weathering ophiolite range in a densely vegetated, tropical biome. Uranium-thorium radiometric dating suggests that the Zambales ophiolite complex is approximately 40 Myr old, placing formation of the oceanic crust in a nascent island arc during Eocene seafloor spreading (Hawkins and Evans, 1983; Abrajano et al., 1988). Average δD_CH4_ values of −136‰ suggest thermogenic production of CH_4_ at temperatures ~110–125°C, consistent with active serpentinization (Abrajano et al., 1988). Previous work at Los Fuegos Eternos (approximately 50 km southwest of Manleluag) suggests that serpentinization in Zambales produces an average ratio of 55:42 CH_4_:H_2_ (Abrajano et al., 1988). In general, previous work has suggested that methane and molecular hydrogen in Zambales gas seeps is originating from the serpentinization reaction involving ultramafic rock and meteoric (not mantle-derived) water (Abrajano et al., 1988, 1990).

There are numerous publications to date investigating the geobiology of ultramafic systems (Barnes et al., 1978; Blank et al., 2009; Etiope et al., 2011; Brazelton et al., 2013; Cardace et al., 2013; Suzuki et al., 2013; Szponar et al., 2013; Sánchez-Murillo et al., 2014; Meyer-Dombard et al., 2015). However, tropical serpentinizing seeps are relatively scarce in the literature (Schrenk et al., 2013; Sánchez-Murillo et al., 2014). Tropical systems are of particular interest to serpentine-hosted microbial community analysis given the potential for relatively higher influxes of dissolved inorganic and organic carbon via meteoric water input (Abrajano et al., 1990). Investigations of terrestrial serpentinizing seep fluid communities may also have broader interest to Early Earth and astrobiology studies. Oceanic mafic and ultramafic rocks were widespread on Early Earth, and ophiolite terranes have been suggested as particularly compelling analogs for astrobiology studies (Blank et al., 2009). These findings may therefore provide clues to life's earliest origins (Russell, 2003).

## Materials and methods

### Geochemistry

Fluid and solid samples were collected concurrently with samples for DNA analysis to characterize the geochemical environment of the microbial community. Briefly, fluids were collected via peristaltic pump through Sterivex™ filter units (EMD Millipore, Merck KGaA, Darmstadt, Germany) for DIC, DOC, cation/anion, and C and N isotope analysis. Solid material was collected for C and N isotope analysis, freeze dried, and sent to the University of Arizona for analysis by EA-irMS. Dissolved gas was collected in acid-washed, 10 mL glass serum vials sealed with butyl stoppers and flushed with argon gas. Approximately 5 mL of sample fluid was added to the vial, and the gas in the headspace analyzed via gas chromatography (Varian Saturn® 2200 GCMS/MS, Agilent Technologies, Santa Clara, CA, USA) at the University of Chicago. For a detailed explanation of fluid, solid, and gas geochemistry protocols referred to in this manuscript, please see Cardace et al. (2015), and Meyer-Dombard et al. (2015).

### Field site

The field site is located at Manleluag Spring National Park (N15°42′16″ E120°16′52″) in Mangatarem, Pangasinan province, the Philippines, approximately 270 km northwest of Manila. The site is part of the Acoje block of the middle ultramafic belt of the Zambales ophiolite range (Abrajano et al., 1990). The climate is tropical, with average annual temperatures of 27.4°C, and average annual rainfall of 2223 mm. For a detailed analysis of the aqueous geochemistry and bioenergetics of the system, please see Cardace et al. (2015).

**Figure 1:**
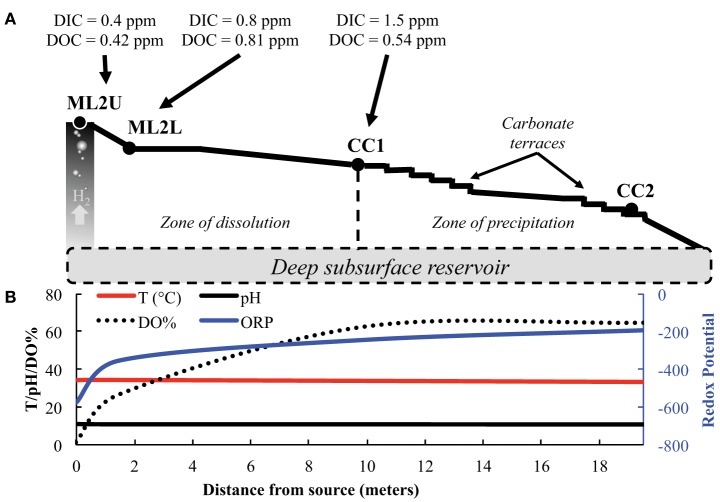
**Manleluag source and outflow channel site sampling regime**. **(A)** Samples were collected from Manleluag Seep 2 Upper (ML2U), Lower (ML2L), and Caustic Cascade 1 (CC1). H_2_ degasses from the serpentinizing fluid as it approaches ML2U. Carbon dioxide in the atmosphere dissolves as inorganic carbon (DIC) along the outflow channel. Leaf litter and insects, etc… contribute to dissolved organic carbon (DOC) along the outflow channel. Scale bar denotes distance from source in meters. **(B)** Selected geochemical data from Manleluag; temperature, pH, and DO% are presented on the primary y-axis, ORP (oxidative-reductive potential) on the secondary y-axis, and distance from the source, in meters, on the x-axis. Please see Cardace et al. (2015), for a detailed analysis of geochemical parameters.

Samples were collected in 2012 and 2013 along the outflow channel of a serpentinizing seep named Manleluag 2 (ML2) (Figure 1). Fluid and solid samples for molecular microbial community analysis, microscopy, and geochemistry were collected at three locations: (1) the source pool (ML2U); (2) in a larger, meter-wide pool below the source (ML2L); and (3) at carbonate terraces approximately 10 meters downstream (CC1). Solid samples and meter readings were collected from a fourth location (CC2) (Figure 1). CC1 and CC2 are subsets of the location described as “ML3” in Cardace et al. (2015). Samples for DNA extraction were collected at ML2U, ML2L, and CC1 for high-throughput sequence analysis.

### SEM-EDX

A Hitachi S-3000N Variable Pressure Scanning Electron Microscope (SEM; Hitachi High-Technologies Corporation, Tokyo, Japan) was used to confirm the presence of microbes in solid samples. The SEM is equipped with an Oxford INCA energy dispersive X-ray spectroscopy (SEM-EDX), with a light element X-ray detector (Oxford Instruments, Tubney Woods, Abingdon, Oxfordshire, UK). Samples were freeze-dried in a Labconco Freeze Dry System/Freezone 4.5 (Labconco, Kansas City, MO, USA) and attached to aluminum stubs with carbon tape (Electron Microscopy Sciences, Hatfield, PA, USA). Samples were uncoated and unpolished. Images were acquired with the backscattered electron detector using an accelerating voltage of 10–20 kV in variable pressure mode (ranging from 10 Pa to 70 Pa pressure). Weight percent of elements were measured with the X-ray detector at a standard working distance of 15 mm.

### Description of carbonates

The following terminology is used here to describe the travertine carbonate deposits at Manleluag: *terrace* refers to meter-scale structures which form as water pools in the dam created by precipitating carbonate; *rimstone dam* refers to the exposed rim of the terrace; *terracettes* refer to cm-scale features, and *microterracettes* to mm-scale features on faces of rimstone dams.

Calcium carbonate solubility was calculated via fluid chemistry data from ML2U, ML2L, and CC1. Carbonate speciation was adjusted for the pH at each site, but calculations were otherwise performed at standard temperature and pressure. The total dissolved inorganic carbon (DIC) represents the total carbon in the system (C_T_). See the Supplemental Material for a complete account of equations.

### DNA extraction

Samples for DNA analysis were collected in sterile Whirl-Pak bags (Nasco, Fort Atkinson, WI, USA) and transported on ice. Samples were stored in a −20°C freezer until processing. Environmental DNA was extracted with the PowerBiofilm™ DNA Isolation Kit (MO BIO Laboratories, Inc., Carlsbad, CA, USA) in replicate, and samples were pooled, concentrated, and purified using the QIAquick® PCR Purification Kit (QIAGEN, Germantown, MD, USA).

### Illumina MiSeq sequencing

Samples were sequenced on the Illumina MiSeq platform (Illumina, Inc., San Diego, CA, USA) at Argonne National Laboratory's Institute for Genomics and Systems Biology Next Generation Sequencing Core (IGSB-NGS) (Argonne, IL, USA). Region-specific primers were used to amplify the V4 region (515F-806R) of the 16S rRNA (rDNA) gene. These primers are optimized to be nearly universal across bacterial and archaeal domains, but provide poor coverage of eukaryal sequences (Walters et al., 2011). Paired-end reads were analyzed using the QIIME pipeline (Caporaso et al., 2010a). Briefly, sequences were demultiplexed, barcode-trimmed, and quality filtered. Operational taxonomic units (OTUs) were selected with a 97% identity threshold using the UCLUST algorithm (Edgar, 2010). Representative sets of sequences were selected for taxonomy assignment using the RDP classifier (Wang et al., 2007; McDonald et al., 2012; Werner et al., 2012). Sequences were aligned with PyNAST against the Greengenes core set default reference alignment (DeSantis et al., 2006; Caporaso et al., 2010b). The aligned sequences were filtered for gaps and non-conserved positions, and the filtered alignment used to produce phylogenetic trees using the FastTree 2.1.3 algorithm in QIIME (Price et al., 2010). Rarefaction of sequence data was conducted to calculate alpha and beta diversity. Faith's phylogenetic diversity, Chao1, and observed species counts were calculated for each sample. Weighted and unweighted UPGMA diversity distance matrices were also calculated for each sample, and a consensus hierarchical dendrogram was constructed (Lozupone and Knight, 2005; Vazquez-Baeza et al., 2013).

### Nucleotide sequence accession number

Nucleotide sequence data have been submitted to the NCBI SRA database under project accession number PRJNA269301.

## Results

### Site description

**Figure 2:**
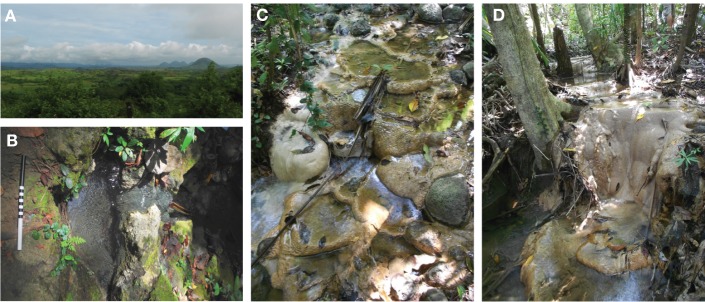
**Site location**. **(A)** The hilly terrain of the Zambales ophiolite range in Luzon, Philippines. **(B)** The source seep at Manleulag Spring National Park (scale bar = 30 cm). **(C)** Terraces and rimstone dams, 9.9 m from source. **(D)** A large accretion of terraces on tree roots and fallen branches, 19.5 m from source.

**Figure 3:**
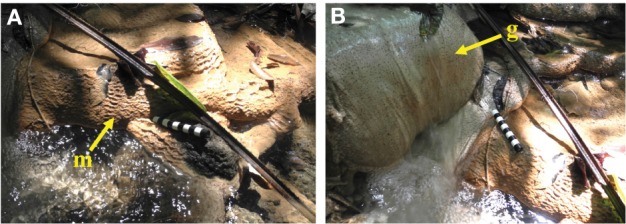
**Terrace formation at CC1**. **(A)** Terraces range in scale from mm (microterracette, *m*) to meters. Microterracettes form evenly spaced ridges along the rims of the larger, cm-sized terracettes. Scale bar equals 10 cm. **(B)** Serpentinizing fluid flowing over gabbro forms a mound-like feature (*g*). Scale bar equals 10 cm.

The serpentinizing seep is located on a densely vegetated slope in the eastern Zambales Mountains (Figure 2A). The seep's surface expression creates a small, deep pool in the gabbro bedrock (Figure 2B). The outflow cascades over the bedrock and forms a wide, shallow pool (Figure 2B). Approximately 10 meters along the outflow channel, precipitation of calcium carbonate creates meters of cascading terraces (Figures 2C,D, 3). Temperature and pH decrease slightly from the source pool at ML2U to the carbonate terraces at CC2 (Table 1). The oxidation-reduction potential (ORP) is lowest at the source, and becomes more positive with distance from the source (Table 1).

**Table 1:** **Selected geochemical data collected from Manleluag Spring National Park**.

**Site characterization**					**DIC**		**DOC**		**Total carbon (C_T_) of solids**	
	**Meters from source**	**T (°C)**	**pH**	**ORP**	**C (ppm)**	**δ^13^C_VPDB_ (‰)^a^**	**C (ppm)**	**δ^13^C_VPDB_ (‰)**	**C (%)**	**δ^13^C_VPDB_ (‰)**
ML2U	0	34.5	10.9	−579.8	0.47	−16.5	0.42	−26.5	0.72	−26.5
ML2L	1.45	34.3	10.9	−354.7	0.82	−15.1	0.81	−25.5	0.44	−24.9
CC1	9.86	33.8	10.8	−245.0	1.53	−18.7	0.54	−26.8	11.5	−19.0
CC2	19.51	33.3	10.8	−193.5	nd^b^	nd	nd	nd	nd	nd

a *VPDB, vs. Vienna Pee Dee Belemnite δ^13^C standard*.

b *nd, no data collected*.

The concentration of DIC increases three-fold from the source to the terraces (Table 1). Additional organic input enters the stream in the form of soil, vegetation, and insects (Figure 1) and DIC fluctuates along the outflow channel (Table 1). Weight percent of carbon in solids decreases initially from a value of 0.72 wt.%, and then increases to 11.5 wt.% (Table 1). The isotopic composition of carbon (as DIC) is increasingly depleted as distance from the source increases. In solids, isotopic composition of carbon (total, C_T_), is enriched with distance from the source. The isotopic composition of DOC is relatively constant in the stream. At the source of the seep, the isotopic composition of the C_T_ in solids and that in the DOC are equally depleted (Table 1).

### Carbonate solubility

**Figure 4:**
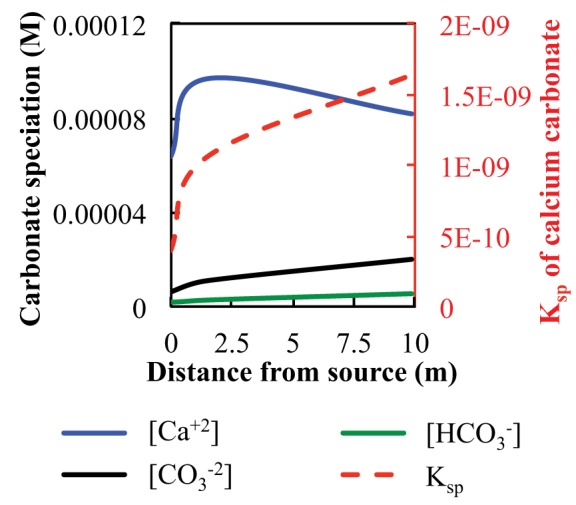
**Fluid carbonate species concentration, in M, and calculated K_sp_ of CaCO_3_ at Manleluag, plotted on a secondary axis (dashed line, red)**.

**Figure 5:**
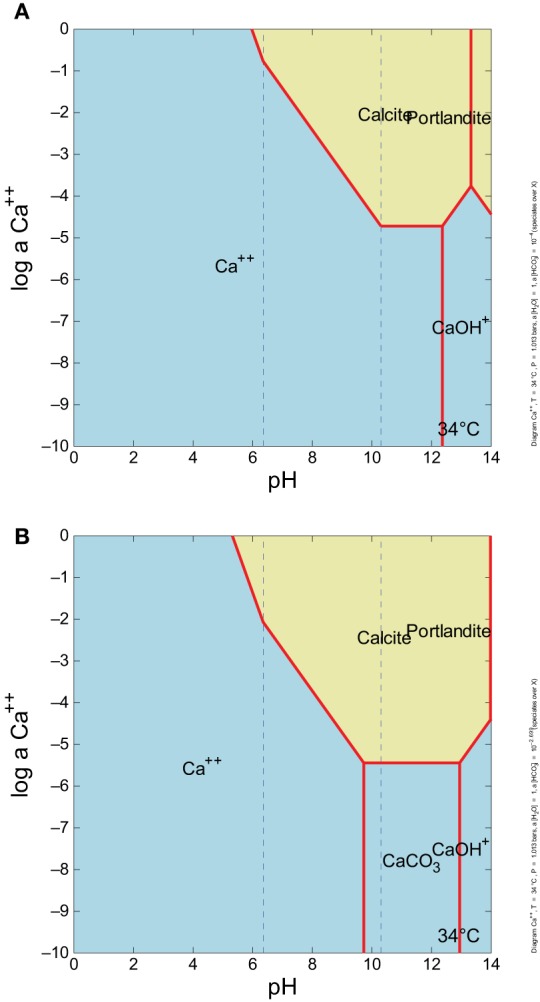
**Ca^+2^ activity vs. pH, with **(A)** ~0.0001 m HCO^−^_3_ (which is equivalent to 1 ppm total C as DIC), and **(B)** ~0.002 m HCO^−^_3_, about what is in surface water**. There is no CaCO_3_ aqueous complex field in the **(A)**, but there is in **(B)**. As CO_2_ increases, the aqueous CaCO_3_ complex field forms, and the stability field of Portlandite (CaOH_2_) shifts to the right.

Carbonate solubility was calculated for fluids at the source (ML2U), and downstream sample sites (ML2L and CC1) based on DIC field measurements (Table 1, Figure 4). The solubility product constant (K_sp_) increases as distance from the source increases. Calculated values for CO^−2^_3_ and HCO^−^_3_ in fluids increase with distance from the source. Measured values for Ca^+2^ increase initially, then decrease with distance from source (Figure 4). The stability field of CaCO_3_ shifts by an order of magnitude as CO^−2^_3_ ion availability increases in the system via atmospheric exposure (Figure 5).

### SEM-EDX

**Figure 6:**
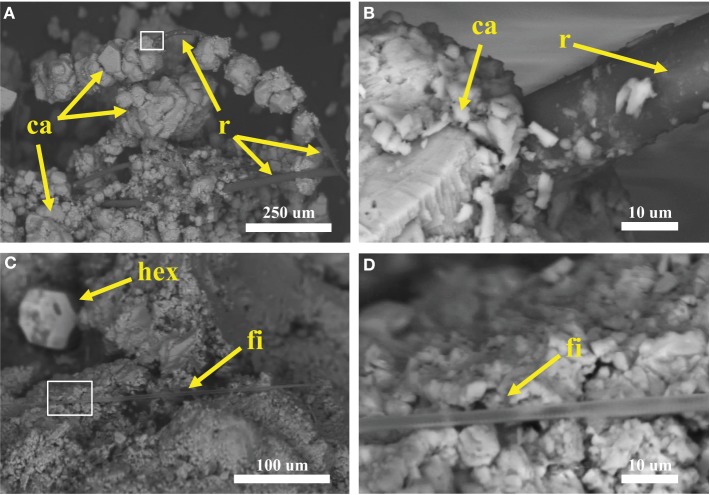
**Scanning electron microscopy (SEM) of samples collected at Manleluag Spring National Park**. **(A)** Terrace samples consist of rhomboid calcite crystals (*ca*), shown here precipitating on organic matter, likely a plant root (r). **(B)** Close-up of box in **(A)**. **(C)** Putative cyanobacterial filament (*fi*) interbedded with calcite crystals (note hexagonal crystal, *hex*, in background). **(D)** Close-up of box in **(C)**.

**Figure 7:**
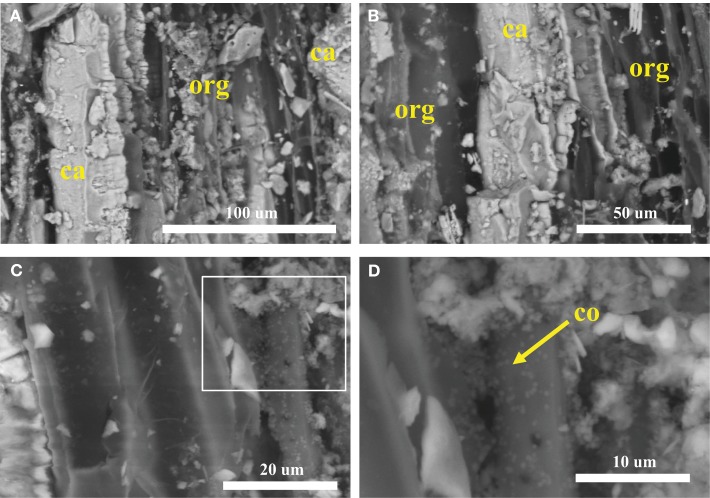
**SEM of a plant root carbonate casing, viewed in cross section**. **(A,B)** Cross sectional view of bands of organic rich (*org*) and calcium-rich (*ca*) material. **(C)** Biofilm layer. **(D)** Close-up of box in **(C)** containing putative coccoid cells.

Plant roots and microbial filaments are confirmed in the carbonate samples collected from Manleluag (Figure 6). Elemental analysis of solids at CC2 indicates that the weight percent (wt.%) of carbon varies between calcium-rich (*ca*, 38.2 wt.% C) and organic-rich (*org*, 36.16 wt.% C) bands in a cross section of a carbonate encrustation collected from a small root in the outflow channel (Table 2, Figure 7).

**Table 2:** **Elemental weight% of solids at Manleluag**.

**Wt.%**	**Source sed**.	**Ca-rich**	**Org. rich**	**Crystal rhomb**.	**Root**
C	24.4	1.72	36.2	4.86	52.4
O	32.2	40.9	41.0	46.5	30.1
Mg	-	2.85	4.4	2.16	1.2
Al	1.5	3.6	2.85	3.64	0.93
Si	2.5	8.88	7.11	6.65	2.22
S	-	-	0.25	-	-
Ti	18.6	-	-	-	-
Mn	0.84	-	-	-	-
Ca	0.37	38.2	7.38	32.7	12.1
Fe	19.55	3.92	0.89	3.54	1.09

**Figure 8:**
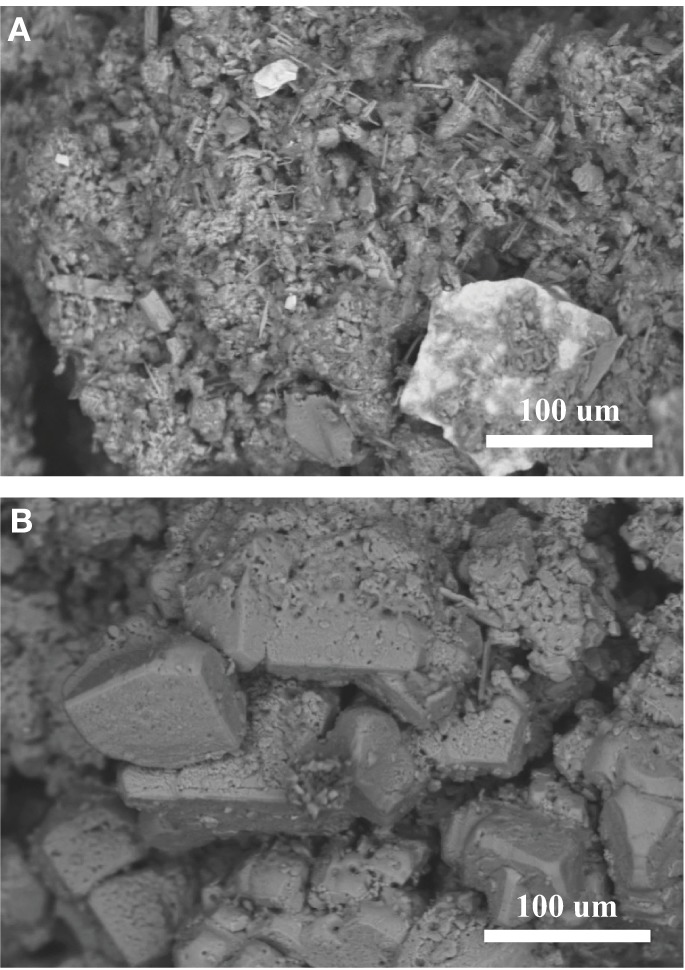
**SEM of sediments collected from Manleluag**. **(A)** Sediment from the bottom of a pool formed by a rimstone dam at CC1. Lithified pool bottoms show evidence of secondary precipitation in the form of aragonitic needles and bushes. **(B)** Rhombohedral crystals from the terrace rim.

Sediment collected at the source seep contains iron- and titanium-rich minerals, (Table 2) and exhibits less carbonate than sites farther downstream. Crystallization of calcium carbonate is variable based on depositional environment; a lithified pool bottom (Figure 8A) shows secondary aragonitic precipitation and reworking of crystals, while the rhombohedral crystals on the terrace rim (Figure 8B) are much larger and appear less degraded/weathered.

Solids at all sites demonstrate low elemental wt.% magnesium (Table 2). Sulfur was only detected in the organic-rich layers of the encrusted root carbonate (Table 2). Titanium and manganese were only detected in source sediment. Iron, aluminum, and silica were detected in all samples, to varying degrees (Table 2). The highest elemental wt.% iron is observed in sediment collected from the source (ML2U).

### 16S rRNA gene amplicon sequencing

16S rRNA gene amplicon sequencing analysis yielded a total of 148906 reads with an average sequence length of 245.35 (±13.65). Samples were filtered to a minimum OTU count of 50, and after filtering ML2U contained 42339 sequences, ML2L contained 41419 sequences, and CC1 contained 65168 sequences.

### Alpha and beta diversity

**Figure 9:**
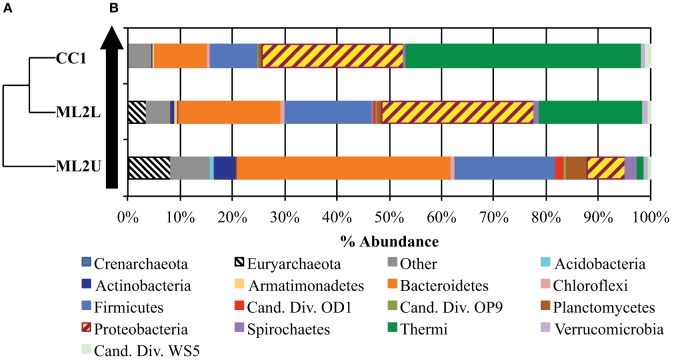
**(A)** UPGMA-clustered dendrogram demonstrates relatedness between samples. **(B)** Histogram of OTUs present at least 50 times in the OTU table, separated by phylogenetic assignment to the Phylum level. The black arrow represents direction of flow in the seep.

Rarefaction was performed to a maximum depth of 65,000 sequences/sample and 10 replicates per iteration. Analysis of within-sample alpha diversity at Manleluag suggests that the microbial community at ML2U exhibited the highest biodiversity in all tested metrics (Supplemental Figures 1, 2). A comparison of observed species and Chao1 diversity indices suggests under-sampling of the communities at Manleluag. UPGMA-clustered hierarchical dendrogram analysis indicates sample relatedness; ML2L and CC1 cluster more closely than ML2U (Figure 9).

### Taxonomy assignment and OTUs

Taxon abundance, represented as a percent of the total sequence reads, indicates that microbial community composition shifts downstream from the source as microbes adapt to an influx of oxygen, DIC, and sunlight. The most abundant taxa (OTUs that are present in the OTU table at least 50 times) varied by location. At ML2U, *Methanobacterium* spp. comprised 8.1% of total sequence reads (Figure 9). The most abundant taxon identified is Bacteroidetes (40.7% total sequence reads). Firmicutes encompass the second largest identified phyla (19.1%). At ML2L, Proteobacteria and Thermi become increasingly abundant, and by CC1, Thermi, comprised almost entirely of *Meiothermus* spp., represents 45% of the total microbial community (Figure 9).

**Figure 10:**
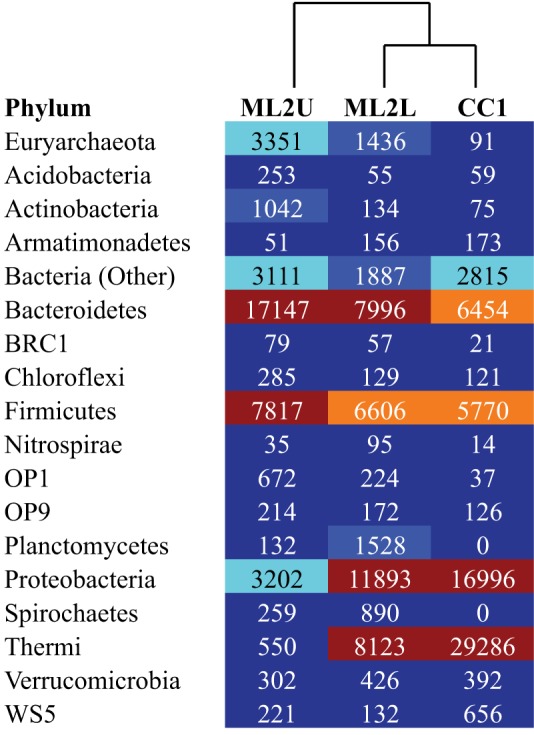
**Heatmap of OTUs separated to the Phylum level, with UPGMA-clustered dendrogram**. Colors correlate to OTU density (dark red is the highest density, dark blue the lowest).

An OTU heatmap illustrates OTUs (phylotypes) by sample location (Figure 10). The phyla Bacteriodetes, Firmicutes, and Euryarchaeota are the most abundant taxa at ML2U. Bacteroidetes are also present in decreasing orders of abundance at ML2L and CC1 (Figure 10). Sequences related to the phylum Thermi, nearly absent at the source, increases downstream to become the most dominant phylotype at CC1 (Figure 10).

A phylogenetic tree was constructed using the FastTree algorithm to demonstrate the relatedness between phylotypes (Supplemental Figure 3).

### Comparison to other serpentinizing studies

**Figure 11:**
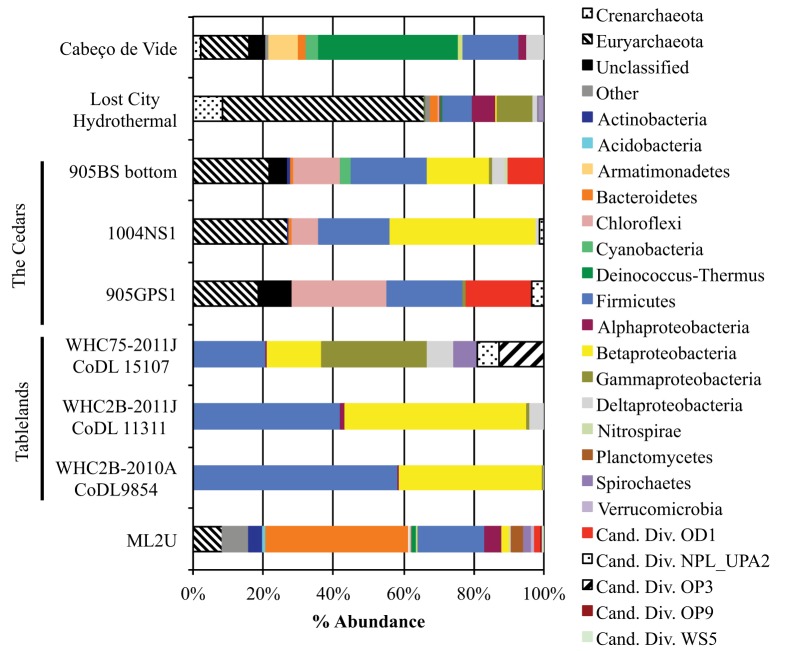
**Comparison between phylotypes from the source at Manleluag (ML2U) and results from studies of other serpentinizing systems, separated by Phylum (and class, in the Phylum Proteobacteria)**. Examples include terrestrial serpentinizing seeps from Tablelands Ophiolite Complex in Newfoundland, Canada (Brazelton et al., 2013) and the Cedars in the Coast Range Ophiolites, USA (Suzuki et al., 2013). Also included are examples from 35-year old carbonates at Lost City Hydrothermal Field (Brazelton et al., 2010) and a subterranean serpentinizing aquifer, Cabeço de Vide, in Portugal (Tiago and Veríssimo, 2013).

The source microbial community was compared to taxon abundance data from other serpentinizing seep studies (Figure 11; Brazelton et al., 2010, 2013; Suzuki et al., 2013; Tiago and Veríssimo, 2013). The sediment at ML2U shares similar taxa with seep sources from other terrestrial serpentinizing seeps at the Tablelands in Newfoundland, Canada, and the Cedars, California, USA (Brazelton et al., 2013; Suzuki et al., 2013). Firmicutes and Proteobacteria were common to all seep locations, and Bacteroidetes were present in all but two (WHC2B-2010A CoDL9854 and 905GPS1) of the serpentinizing seeps (Figure 11).

## Discussion

Microbial community analysis via high-throughput 16S rRNA gene amplicon sequencing reveals that the deep subsurface microbial community at Manleluag responds to surface exposure with decreasing biodiversity, which is directly correlated to increased distance from the source. Meters of carbonate terraces, created by precipitation of Ca^+2^ from the alkaline fluids and CO^−2^_3_ derived from atmospheric CO_2_, provide microbial habitats and record physical and geochemical evidence of microbial activity. These traits suggest that terrestrial serpentinizing ecosystems may be particularly compelling analogs for astrobiology studies.

### Carbonate dissolution and precipitation

The precipitation and dissolution of CaCO_3_ is dependent on the environmental geochemical, thermodynamic, and kinetic factors in any given system. At Manleluag, DIC increases gradually over the sampled transect, and precipitation of CaCO_3_ begins to occur as DIC concentrations reach 1.53 ppm in the outflow channel. (Table 1, Figure 4). The solubility constant (K_sp_) of CaCO_3_ increases rapidly with increasing concentrations of DIC, which at pH ~11 is mostly available as CO^−2^_3_ (Figure 4). The elevated Ca^+2^ near the spring source [at about 3 ppm Ca^+2^ or log *a*Ca^+2^ ~ (−4.1); see Cardace et al., 2015] suggests that calcite precipitation is near the phase boundary between Ca^+2^(aq) and CaCO_3(s)_, inhibited in a sense until more bicarbonate is present (Figure 5A). At high dissolved CO_2_ levels, the Ca^+2^ is at about the same concentration, and squarely in the CaCO_3(s)_ field (Figure 5B).

Fine solids of CaCO_3_ may be forming at the source of the serpentinizing fluid, though are difficult to observe (Figure 2B). The K_sp_ of CaCO_3_(Equation 3) may increase rapidly with increasing distance from the source, given effects of partial pressure of CO_2_ and temperature (Morse and Arvidson, 2002). Temperature and the concentration of Ca^+2^ ions are relatively constant down gradient; however, precipitation becomes thermodynamically inevitable (the reaction quotient, *Q*, of serpentinizing fluids > K_sp_, for the reaction CaCO_3_ → CO^−2^_3_ + Ca^+2^), as the concentration of available CO^−2^_3_ increases due to fluid equilibration with the atmosphere (Figure 4).

The isotopic composition of DIC becomes increasingly depleted as distance from the source increases, suggesting that isotopically lighter atmospheric C is dissolving into the fluid. In the solid sediments and biofilms collected, isotopic composition of total carbon (C_T_) is enriched with increasing distance from the source, while at the source the isotopic composition of C_T_ is equally depleted as the DIC (Table 1). This may indicate that the C_T_ of solids collected at the source originates from the DOC in the source fluid, suggesting biological assimilation in what resembles a closed-loop ecosystem (i.e., the deep subsurface). Farther downstream at site CC1, the isotopic composition of the C_T_ in solids approaches the DIC values, again indicating that CO_2_ from the atmosphere is dissolving into the serpentinizing fluid and precipitating as CaCO_3_, and potentially including influence from microbial carbon fixation and heterotrophy.

### SEM-EDX

SEM results (Figures 6–8) demonstrate that traces of microbial activity may be recorded in precipitated calcium carbonate triggered by serpentinizing fluid exposure to surface conditions. Carbonate-associated biofilms exhibit morphological variation based on location in the outflow channel (Figures 6–8). At CC2, the biofilms are ordered by precipitation cycles, creating bands of alternating organic-rich and mineral-rich material. EDX analysis indicates that carbon abundances vary in organic-rich layers vs. organic-poor calcium carbonate layers (Figure 7). Sulfur was only detected in the organic-rich layers, potentially suggesting sulfur cycling occurs in this layer. Given these data, it appears that at Manleluag, geochemical conditions in the serpentinizing fluid may preserve microbial chemical traces that are in part detectable with X-ray spectroscopy (Table 2, Figure 7). Further investigation via *in situ* isotopic measurements would be highly beneficial in ascertaining the biogenicity of these structures (Blank et al., 2009).

In general, terraces appear to preserve microbial traces more conservatively than the lithified pool bottoms, which demonstrate crystal degradation and secondary precipitation of aragonitic needles on calcite rhomboids (Figure 8). The dissolution and re-precipitation occurring in pool bottoms may re-work many physical and chemical traces of past microbial activity. These results suggest that microbial traces may be preferentially preserved in air-water interface depositional facies, such as terraces. Therefore, terraces, and not pool bottoms, may serve as more appropriate targets for analog studies.

### Microbial transmission from subsurface to surface

The taxonomic assessment indicates that phylotypes of deep subsurface taxa continue to be present in 16S rRNA gene amplicon sequences, to the farthest extent measured (CC1). Alpha diversity indices (measuring diversity within the sample) suggest that the source site (ML2U) is the most biodiverse; the dynamic subsurface-surface interface appears to support a wide range of facultative and obligate anaerobes and aerobes (Supplemental Figures 1, 2). As an interface between the subsurface and surface environments, the pool associated with the seep orifice exemplifies an ecotone that shares characteristics of each ecosystem while also having characteristics unique to itself. Samples collected at CC1 demonstrate the least biodiversity, with nearly 45% of the sequence alignments affiliating with *Meiothermus* spp. This is in contrast to other terrestrial serpentinizing seep studies conducted at the Tablelands ophiolite, where TRFLP analysis suggested the highest biodiversity was found in the well-mixed freshwater end member (Brazelton et al., 2013). In Manleluag, pH is relatively constant ~10.8, and while dissolved oxygen becomes increasingly abundant, the concentration of bioavailable DIC remains very low. Typical of the high pH of serpentinizing ecosystems, CO^−2^_3_ concentration is an order of magnitude higher than HCO^−^_3_ in Manleluag, and this appears to suppress the diversity and abundance of photoautotrophs (Suzuki et al., 2013). The abundance of CO^−2^_3_ and Ca^+2^ ions causes precipitation on biological surfaces (Figures 6A,B, 3). *Meiothermus* spp. may have some propensity for survival in this uniquely challenging environment, given its preference for alkaline environments and ability to form biofilms and anchor itself to surfaces, thus preventing being swept downstream (Kolari et al., 2003).

*Sphingomonas* spp., have been isolated from deep aquifers despite classification as strict aerobes (Fredrickson et al., 1999). Lautenschlager et al. (2014) demonstrated that *Sphingomonas* spp. are capable of oxidizing a wide range of aromatic compounds and produce acetate and methane from carbon oxidation, and calculations suggest that respiration rates are extremely low based on geochemical modeling of the environment. Similarly, this may explain the abundance of other aerobic heterotrophs at the source, such as *Meiothermus* spp., which may have been respiring very slowly in the deep subsurface but now thrives at the surface where oxygen is abundant (Tindall et al., 2010).

Metabolic function in Manleluag is inferred by the known metabolisms of closest neighbors of the taxa identified above, and via bioenergetics calculations based on geochemical profiling of the system (Cardace et al., 2015). These putative metabolisms may include: hydrogen oxidation, fermentation, H_2_-associated sulfur cycling, methanogenesis, methanotrophy, nitrogen fixation, ammonia oxidation, denitrification, nitrate respiration, methylotrophy, carbon monoxide respiration, and ferrous iron oxidation. The most abundant taxa identified across all three sites at Manleluag include: *Sphingomonas* spp., an aromatic carbon degrader (Lautenschlager et al., 2014); *Meiothermus* spp., an aerobic heterotroph related to Deinococcus-Thermus group taxa also detected at Lost City Hydrothermal Field and the Cabeço de Vide aquifer in Portugal (Brazelton et al., 2010; Tiago and Veríssimo, 2013); anaerobic, hydrogen oxidizing Bacteriodales (Blank et al., 2009; Tiago and Veríssimo, 2013); heterotrophic Coriobacteriaceae which align with taxa also found in the Cabeço de Vide aquifer, and may represent a surface mixing taxon (Tiago and Veríssimo, 2013); *Thiobacillus* spp., an obligate chemolithoautotroph known to oxidize hydrogen, reduced sulfur and ferrous iron (Kelly and Wood, 2000); *Methanobacterium* spp., an anaerobic methanogen typically found in deep subsurface samples and terrestrial serpentinizing seep point sources (Moser et al., 2005; Blank et al., 2009); *Hydrogenophaga* spp., a hydrogen-oxidizer typical to serpentinizing ecosystems (Brazelton et al., 2010, 2013; Suzuki et al., 2013); Xanthomonadaceae, a carbon degrader hypothesized to belong to a deeply branching clade in the Gammaproteobacteria (Anzai et al., 2000), and Rhodobacteriaceae, chemoorganotrophs that have been detected in other serpentinizing environments (Brazelton et al., 2010; Jungbluth et al., 2014).

“Rare” taxa (less than 3% relative abundance) include the ammonia-oxidizing archaea Thaumarchaeota, (Spang et al., 2012) and *Exiguobacterium*, a facultative anaerobe capable of survival in a broad range of microbial environments, detected at the source seep (Vishnivetskaya et al., 2009). *Exiguobacterium* sp. AB2, a haloalkaliphilic strain of *Exiguobacterium*, was recently isolated from samples also collected in the Manleluag seep (Cabria et al., 2014). Other rare taxa include *Sporosarcina*, some of which are capable of microbially induced calcite precipitation and cementation via hydrolysis of urea (Yoon et al., 2001); *Alkaliphilus*, an extreme alkaliphile isolated from an ultra-deep gold mine in South Africa (Takai et al., 2001); *Dethiobacter*, an alkaliphile that grows chemolithoautotrophically using H_2_ as an electron donor and thiosulfate, elemental sulfur, and polysulfide as electron acceptors (Sorokin et al., 2008); *Desulfonatronum*, an alkaliphilic, sulfate reducing bacterium (Pikuta et al., 2003); *Mycobacterium*, a ubiquitous, alkaline-tolerant genus (Bland et al., 2005); *Roseococcus*, an obligately aerobic, facultative alkaliphilic photoheterotroph (Boldareva et al., 2009); *Roseomonas*, representatives of which exhibit alkalitolerance and thermotolerance (Dong et al., 2014); *Ramlibacter*, a deeply branching clade isolated from weathered pyroxene, chromite and secondary calcite crystals of a meteorite collected in 1931 in Tunisia (Heulin et al., 2003); and *Deinococcus*, a bacterial genus known for its resistance to dessication, UV and ionizing radiation (Makarova et al., 2001).

### Comparison to other sites

The source seep at Manleluag (ML2U) exhibited shared phylotypes with several terrestrial, sub-seafloor, and deep aquifer serpentinizing ecosystems (Brazelton et al., 2010, 2013; Suzuki et al., 2013; Tiago and Veríssimo, 2013). The presence of Firmicutes, particularly Clostridia, was universal to all data sets, including this study (Figure 11). ML2U exhibited percent abundances of Firmicutes similar to those documented at the Cedars (Coast Range Ophiolite, California, USA) and a pH 11.2 serpentinizing seep in the Tablelands, Newfoundland, Canada (WHC75-2011J CoDL 15107; Brazelton et al., 2013; Suzuki et al., 2013). Representatives of the methanogenic *Methanobacterium* were also common to all compared serpentinizing studies (except for the Tablelands, which did not include archaeal sequence data; Brazelton et al., 2013).

Bacteroidetes were also common (in all but two of the referenced sequence data sets), and is the most abundant taxon at ML2U (Figure 11). Betaproteobacteria were also frequently observed in serpentinizing sites, particularly the genus *Hydrogenophaga*, which is hypothesized to oxidize hydrogen in serpentinizing ecosystems via mechanisms not entirely understood (Suzuki et al., 2013). The sizeable biogeographical distribution of these taxa supports the hypothesis of an interconnected deep biosphere, either via meteoric water input, through the fractures and pores of the host rock, or a combination of the two. A global picture of the extent and diversity of microorganisms in the deep subsurface is beginning to emerge.

## Conclusions

The microbial community at the Manleluag serpentinizing seep ecosystem demonstrates phylogenetic similarities to other serpentinizing seep ecosystems in a number of different biomes (Brazelton et al., 2010, 2013; Suzuki et al., 2013; Tiago and Veríssimo, 2013). Analysis of the outflow channel generated by the seep suggests a dynamic microbial community response to surface exposure. Surface mixing increases the redox potential of the system, thereby increasing the range of metabolic options for microorganisms in the environment. The ecotone environment enhances metabolic diversity, and increasing distance from the source pool results in decreased phylogenetic diversity. The abundance of sequences affiliated with hydrogen-oxidizing bacteria in the outflow channel also increases; this potentially indicates that hydrogen oxidation becomes favorable downstream from the source. Taxa related to heterotrophs predominate, and despite an abundance of sunlight, abundant populations of cyanobacteria were not detected. The lack of photosynthetic metabolisms likely arises from the low DIC concentrations in the serpentinizing fluids. Additionally, the abundance of organic matter (i.e., plant matter, root exudates, insects, etc…) may provide a highly favorable environment for heterotrophic dominance. Metagenomic analysis is currently underway to determine the diversity of putative metabolisms identified in this paper.

The universality of taxa such as Bacteroidetes, Clostridia, and *Hydrogenophaga* suggest that these microbes are ubiquitous in the subsurface environment, though the mode of dispersion is not entirely understood and requires further between- and within-site diversity analyses. Future directions may also include the relationship between increased DIC and DOC at Manleluag in the rainy vs. the dry season, and the possible implications for calcium carbonate precipitate morphology. For example, if seasonal increases in DOC trigger banding of organic-rich layers (Figure 6), then these microstructures may serve as excellent biomarkers in serpentinite-associated carbonates, particularly in tropical environments that experience the same bimodal seasonality (Sánchez-Murillo et al., 2014).

### Conflict of interest statement

The authors declare that the research was conducted in the absence of any commercial or financial relationships that could be construed as a potential conflict of interest.
