# Supplementary material for: Out of the dark: transitional subsurface-to-surface microbial diversity in a terrestrial serpentinizing seep (Manleluag, Pangasinan, the Philippines)
Source: Front Microbiol. 2015 Feb 19;6:44. doi: 10.3389/fmicb.2015.00044 (PMC4333863; doi:10.3389/fmicb.2015.00044)
Supplement: Supplementary file 1 [file DataSheet1.DOCX]

***Supplementary Material***

**­­­­­­­­­Out of the dark: Transitional subsurface-to-surface microbial diversity in a terrestrial serpentinizing seep (Manleluag, Pangasinan, the Philippines)**

**Kristin M. Woycheese^1,^ *, D’Arcy R. Meyer-Dombard^1^, Dawn Cardace^2^, Anacleto M. Argayosa^3^, Carlo A. Arcilla^4^**

^1^Department of Earth and Environmental Sciences, University of Illinois at Chicago, Chicago, IL, USA

^2^Department of Geosciences, University of Rhode Island, Kingston, RI, USA­­

^3^Institute of Biology, University of the Philippines, Diliman, Quezon City, the Philippines

^4^National Institute of Geological Sciences, University of the Philippines, Diliman, Quezon City, the Philippines

*** Correspondence:** Kristin M. Woycheese, Department of Earth and Environmental Sciences, University of Illinois at Chicago, 845 W. Taylor St., M/C 186, Chicago, IL, 60607, USA.

[kwoych2@uic.edu](mailto:kwoych2@uic.edu)

1. **Supplementary Data**

CARBONATE SOLUBILITY CALCULATIONS

The total dissolved inorganic carbon (DIC) represents the total carbon in the system (C_T_).

$$C_{T} \left( DIC \right)= \left[ H_{2}{CO}_{3}^{*} \right]+\left[ {HCO}_{3}^{-} \right]+[CO_{3}^{-2}]$$

Carbonate speciation are calculated from C_T_ (DIC):

$$[H_{2}CO_{3}^{*}] = C_{T} \times\alpha_{0}$$

$$[HCO_{3}^{-}] = C_{T} \times\alpha_{1}$$

$$[CO_{3}^{-2}] = C_{T} \times\alpha_{2}$$

Species Fractionation Calculations:

$$\alpha_{0}={\{1 + K_{1}/[H^{+}] + K_{1}K_{2}/{[H^{+}]}^{2}\}}^{-1}$$

$$\alpha_{1}= {\{ [H^{+}]/K_{1}+ 1 + K_{2}/[H^{+}]\}}^{-1}$$

$$\alpha_{2}= {\{{[H^{+}]}^{2}/K_{1}K_{2}+ [H^{+}]/K_{2}+ 1\}}^{-1}$$

Solubility of Carbonate:

$K_{sp}=[Ca^{+2}][CO_{3}^{-2}]$

First dissociation of carbonic acid:

$$K_{1}=\frac{\left[ H^{+} \right]\left[ \mathrm{HCO}_{3}^{-} \right]}{[H_{2}\mathrm{CO}_{3}^{*}]}={10}^{-6.35}$$

Second dissociation of carbonic acid:

$$K_{2}=\frac{\left[ H^{+} \right][{CO}_{3}^{-2}]}{[{HCO}_{3}^{-}]}={10}^{-10.33}$$

Henry’s law for CO_2_ (pCO_2_ = 10^-3.95^ K_H_ = 10^-1.5^)

$$[H_{2}CO_{3}^{*}] = pCO_{2} \times K_{CO2}$$

Hydration equilibrium constant of carbonic acid:

$K_{h}=\frac{\left[ H_{2}CO_{3}^{*} \right]}{[CO2(aq)]}=1.7 \times{10}^{-3}$

Total Carbon (Dissolved Inorganic Carbon, DIC) is equal to the sum of the dissolved carbonate species:

$$C_{T} \left( DIC \right)= \left[ H_{2}{CO}_{3}^{*} \right]+\left[ {HCO}_{3}^{-} \right]+[CO_{3}^{-2}]$$

Carbonate speciations are calculated from C_T_ (DIC):

$$[H_{2}CO_{3}^{*}] = C_{T} \times\alpha_{0}$$

$$[HCO_{3}^{-}] = C_{T} \times\alpha_{1}$$

$$[CO_{3}^{-2}] = C_{T} \times\alpha_{2}$$

Species Fractionation Calculations:

$$\alpha_{0}={\{1 + K_{1}/[H^{+}] + K_{1}K_{2}/{[H^{+}]}^{2}\}}^{-1}$$

$$\alpha_{1}= {\{ [H^{+}]/K_{1}+ 1 + K_{2}/[H^{+}]\}}^{-1}$$

$$\alpha_{2}= {\{{[H^{+}]}^{2}/K_{1}K_{2}+ [H^{+}]/K_{2}+ 1\}}^{-1}$$

The sum of the species fractionations are equal to one: α_0_+ α_1_+ α_2_= 1.

1. **Supplementary Figures**

**Supplemental Figure 1**: Rarefaction curves for Manleluag samples. Average Faith’s phylogenetic alpha diversity indices versus sampling depth per sample.

**Supplemental Figure 2**: Average alpha diversity indices (observed species and Chao1) versus reads per sample.

**Supplemental Figure 3**: Phylogenetic assignment represents relatedness between sequences at Manleluag. A diverse assemblage of Proteobacteria, Firmicutes, and Bacteroidetes were detected in the microbial communities at the source and along the outflow channel. Scale bar equals local support values.
