# Supplementary figures and images for: Out of the dark: transitional subsurface-to-surface microbial diversity in a terrestrial serpentinizing seep (Manleluag, Pangasinan, the Philippines)
Source: Front Microbiol. 2015 Feb 19;6:44. doi: 10.3389/fmicb.2015.00044 (PMC4333863; doi:10.3389/fmicb.2015.00044)

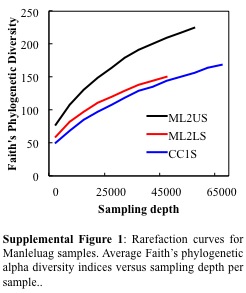

Supplement: Supplementary file 2 [file Image1.JPEG]

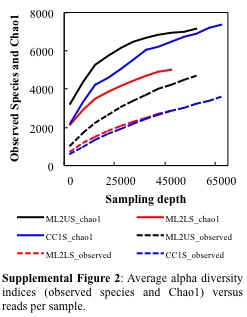

Supplement: Supplementary file 3 [file Image2.JPEG]

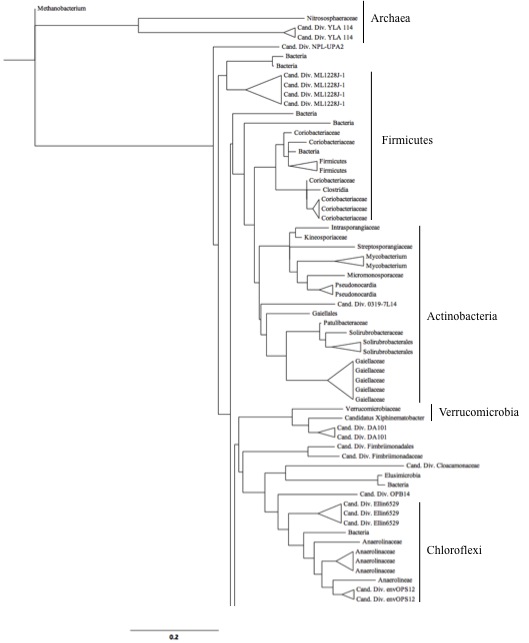

Supplement: Supplementary file 4 [file Image3.JPEG]

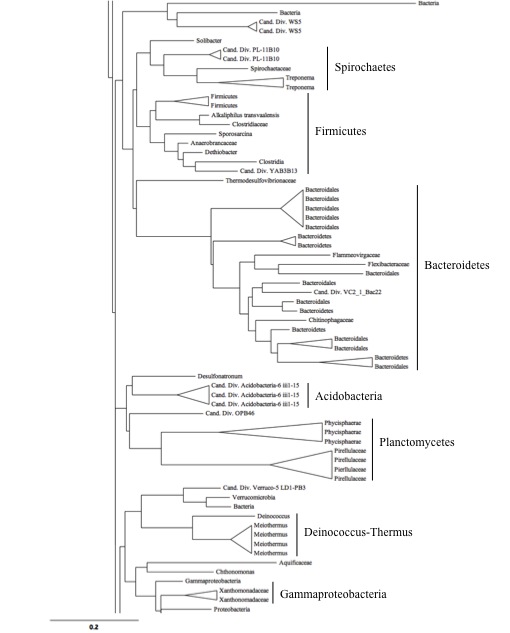

Supplement: Supplementary file 5 [file Image4.JPEG]

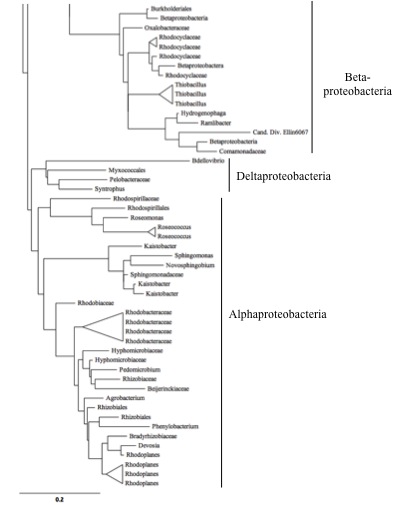

Supplement: Supplementary file 6 [file Image5.JPEG]
